# health service utilization among older population in a terai region of nepal

**DOI:** 10.1093/eurpub/ckac129.650

**Published:** 2022-10-25

**Authors:** B Subedi

**Affiliations:** USAID's SSBH Activity, Abt.Associates.Inc, Rupandehi, Nepal

## Abstract

**Introduction:**

The world is heading towards a larger proportion of older population, indicating an increased risk of diseases, disability, and advanced ageing before death as well as the demand for the health system.

**Methods:**

This study is a community based cross-sectional study, total 329 older people aged 60 years and above were surveyed. Two stage cluster-sampling technique was used. Semi structured questionnaire was used for data collection.

**Results:**

Current study showed that 63.3% of the older population have utilized health services in the past one year. Multivariate analysis showed that, respondents with basic education and secondary education are 0.3(AOR: 0.31, 95% CI: 0.17-0.56) and 0.14 (AOR: 0.14, 95% CI: 0.83-0.26) times less likely to utilize health services, respectively. Similarly, respondents reporting current personnel income above forty thousand are 2.8 (AOR:2.81 95% CI:1.84-4.31) times more likely to utilize health services. Respondents at risk of malnutrition are 2.1(AOR: 2.18, 95% CI: 1.14-4.17) times more likely to utilize health services, similarly undernourished respondents are 3.3 (AOR:3.35,95% CI:1.50-7.51) times more likely to utilize health services as compared to respondents with normal nutritional status. Respondents with chronic disease condition are 11.8 (AOR: 11.89, 95% CI: 6.81-20.74) times more likely to utilize health services as compared to those with no chronic disease condition when holding other variables constant.

**Conclusions:**

There is urgent need to highlight the problems faced by the older population as regards health service utilization and dealing with the identified factors associated with health service utilization among the older population should receive high priority.

**Key messages:**

• Municipality and health facilities should create enabling environment for older population to get necessary health services.

• Awareness program targeting the underprivileged ethnic groups and poor houses are recommended.

